# Dioxin emissions from a municipal solid waste incinerator and risk of invasive breast cancer: a population-based case-control study with GIS-derived exposure

**DOI:** 10.1186/1476-072X-7-4

**Published:** 2008-01-28

**Authors:** Jean-François Viel, Marie-Caroline Clément, Mathieu Hägi, Sébastien Grandjean, Bruno Challier, Arlette Danzon

**Affiliations:** 1 CNRS n° 6249 "Chrono-Environment", Faculty of Medicine, 2, place Saint Jacques, 25030 Besançon cedex, France; 2 Doubs Cancer Registry, Epithelial Carcinogenesis Research Team, University Hospital, 2, place Saint Jacques, 25030 Besançon cedex, France; 3 Computer Medicine Department, University Hospital, 2, place Saint Jacques, 25030 Besançon cedex, France

## Abstract

**Background:**

To date, few epidemiologic studies have examined the relationship between environmental PCDD/F exposure and breast cancer in human populations. Dioxin emissions from municipal solid waste incinerators (MSWIs) are one of the major sources of environmental dioxins and are therefore an exposure source of public concern. The purpose of this study was to examine the association between dioxins emitted from a polluting MSWI and invasive breast cancer risk among women residing in the area under direct influence of the facility.

**Methods:**

We compared 434 incident cases of invasive breast cancer diagnosed between 1996 and 2002, and 2170 controls randomly selected from the 1999 population census. A validated dispersion model was used as a proxy for dioxin exposure, yielding four exposure categories. The latter were linked to individual places of residence, using Geographic Information System technology.

**Results:**

The age distribution at diagnosis for all cases combined showed a bimodal pattern with incidence peaks near 50 and 70 years old. This prompted us to run models separately for women aged 20–59 years, and women aged 60 years or older. Among women younger than 60 years old, no increased or decreased risk was found for any dioxin exposure category. Conversely, women over 60 years old living in the highest exposed zone were 0.31 time less likely (95% confidence interval, 0.08–0.89) to develop invasive breast cancer.

**Conclusion:**

Before speculating that this decreased risk reflects a dioxin anti-estrogenic activity with greater effect on late-onset acquired breast cancer, some residual confounding must be envisaged.

## Background

Established risk factors for breast cancer are hormonally mediated: age, family history of breast cancer, early menarche, late menopause, late first full-term pregnancy or nulliparity, breast density, and benign breast disease [1,2]. But taken together, these well-established risk factors account for only about half of all breast cancer cases [3]. Considerable interest has therefore recently focused on environmental contaminants having the potential to affect breast cancer risk, although explicit environmental links to this disease are still limited.

Dioxin is the name given to two classes of organochlorine compounds; 75 polychlorinated dibenzo-*p*-dioxins (PCDD) and 135 polychlorinated dibenzofurans (PCDF). Seventeen tetrachloro-substituted congeners are toxic, with 2,3,7,8-tetrachlorodibenzo-*p*-dioxin (TCDD) being the most potent. The U.S. Environmental Protection Agency and the International Agency for Research on Cancer have classified TCDD as a human carcinogen [4,5].

This compound is also known to disrupt multiendocrine pathways in animals at body burdens which are close to those present in the background human population [6]. TCDD elicits a number of anti-estrogenic responses in the female reproductive tract, including the impairment of mammary gland differentiation (without blocking the response to exogenous estrogen) in the post pubertal female rat [7], the inhibition of estrogen-induced growth of human mammary cells, and carcinogen-induced mammary cancer in rats [8], the suppression of estrogen-dependent development of mammary cancer in mice bearing breast cancer cell xenografts [8,9], and the inhibition of the breast cancer progression [10].

However, accumulating evidence suggests that TCDD also possesses estrogen-like activity. TCCD induces an estrogen-like gene expression profile in the uteri of immature ovariectomized mice in the absence of histopathological or morphological manifestations [11], and mediates the induction of estrogen dependent tumors in rat [12,13].

The opposing actions of TCDD, anti-estrogenic in the presence of estrogen and estrogenic in its absence, suggest that the effects of TCDD may vary depending on developmental stage at exposure [11]. The ability of TCDD to cause ovarian tumors could also dependent on initiation, length of promotion, and age of the animal when exposed and evaluated [13].

To date, few epidemiologic studies have examined the relationship between PCDD/Fs and breast cancer in human populations. Most of these have consisted of occupational cohorts yielding conflicting results [14]. Warner et al. have recently conducted a retrospective cohort study to examine the association between serum TCDD levels and breast cancer risk in women residing around Seveso, Italy, in 1976, at the time of an industrial explosion that resulted in the highest known population exposure to TCDD [15]. Twenty years later, the relative risk for breast cancer associated with a 10-fold increase in serum log_10 _TCDD levels was significantly increased by 2.1-fold (95% confidence interval [CI], 1.0–4.6), while adjusting for established breast cancer risk factors.

Dioxin emissions from municipal solid waste incinerators (MSWIs) are one of the major sources of environmental dioxins and are therefore an exposure source of public concern. Our team recently detected a cluster of non-Hodgkin lymphoma (NHL) in an area that contains a MSWI with high dioxin emission levels (Besançon, France) [16]. We subsequently found a 2.3-fold risk (95% CI, 1.4 – 3.8) for NHL associated with residence in areas classified as highly exposed to dioxin emitted from this MSWI, while adjusting for a wide range of socio-economic characteristics at the block group level [17].

Both the suggestive results by Warner et al. [15], and the availability of a validated dispersion model as a proxy for dioxin exposure, prompted us to carry out a population-based case-control study focusing on breast cancer in the vicinity of this MSWI.

## Methods

### Study area

The MSWI under investigation has been fully described elsewhere [18]. Situated near the southwest boundary of Besançon (4 km from the city center), and put into service in 1971, it had a capacity of 7.2 metric tons per hour. In 1998, approximately 67,000 metric tons of waste were processed there. Some legal guidelines for incinerator emissions have not been followed at this location. For example, in 1997, exhaust gases were not maintained at temperatures of more than 850°C for the legal time (>2 s), allowing dioxins to be emitted. The first time that the dioxin concentration of an exhaust gas was ever measured (in December 1997), it was found to be 16.3 ng international toxic equivalency factor (I-TEQ)/m^3^, whereas the European guide value is 0.1 ng I-TEQ/m^3^. With such conditions, it could be expected that in the last two decades remarkable amounts of PCDD/Fs were released into the environment.

Since a dispersion model provided a reliable proxy for dioxin exposure on the northeast side of the MSWI (simple terrain), but not in the southwest direction (complex terrain) [18], the 90 blocks located southwest of the MSWI were excluded, leaving 590 blocks as the study area.

### Selection of cases and controls

Invasive breast cancer incidence data were provided by the Doubs cancer registry. The latter was established in 1977 and is complete for breast cancer cases, as ascertained by the ratio of the number of deaths to the number of cases registered during 1993–1997, which at 32% (for the Doubs region) is very similar to those reported in other Western countries [19]. Virtually all cases were histologically verified (97%). Cancer registry procedures are approved by the French National Cancer Registry Committee and the National Commission for the Confidentiality of Computerized Data. Women aged 20 years and over, diagnosed during the years 1996 to 2002, and living in the study area at the time of their diagnosis, were included. The cancer registry extracted anonymous data on age and area of residence at time of diagnosis, and histopathologic subtype, using the International Classification of Disease for Oncology, third edition (ICD-O-3): infiltrating duct carcinoma of not otherwise specified (duct NOS; ICD-O-3 code 8500/3), and lobular carcinoma (ICD-O-3 code 8520/3). For the sake of clarity, the less common and the unknown histopathological subtypes (i.e. all other ICD-O-3 breast cancer codes) were aggregated in an "other or unknown" category. All primary invasive breast cancers were therefore included.

We selected female controls from a reliable and accessible database, the 1999 population census. Because of French privacy laws and confidentiality requirements the only individual (and anonymous) data available to researchers are sex, age categories (0–19, 20–39, 40–59, 60–74 and 75+ years), and residence in a given block. The block is the smallest level of geographic resolution in the French census database and is defined only in densely populated areas. Each block is typically a quadrangle bounded by four streets. In the 1999 census, there are 590 blocks in northeast Besançon (averaging 72 female inhabitants aged 20 years and over), aggregated in 42 block groups (averaging 1008 female inhabitants aged 20 years and over). Unfortunately, no other risk factor data at the individual or block level are available to researchers. However, a set of social and demographic information is available for each block group. We randomly selected age-matched population-based controls, according to a 5-to-1 matching procedure.

### Dioxin exposure modeling

Dioxin exposure assessment has been fully described elsewhere [18]. In no instance was actual individual exposure measured but estimated through exposure zones based on predicted ground-level air concentrations. Briefly, we took advantage of a first-generation Gaussian-type dispersion model (APC3 software, Aria Technologies, Colombes, France), allowing the modeling of the transport and dispersion of dioxin emissions from the MSWI. This model was originally developed to predict the future impact of dioxin emissions, both from an old (but renewed) combustion chamber and from a new oven with up-to-date pollution controls. It was not possible to assess past exposure because past dioxin emission rates had not been collected. However, dispersion modeling is heavily influenced by factors that are stable over time (mean meteorological conditions, terrain elevations and stack height). Thus, we assumed that contour shapes, as derived from the prediction model, were reliable estimates of past dioxin deposition profiles and we used dioxin ground-level concentrations as relative figures rather than absolute figures to estimate past exposure. These geographic-based exposure categories have been assessed through PCDD/F measurements from soil samples.

**Figure 1:**
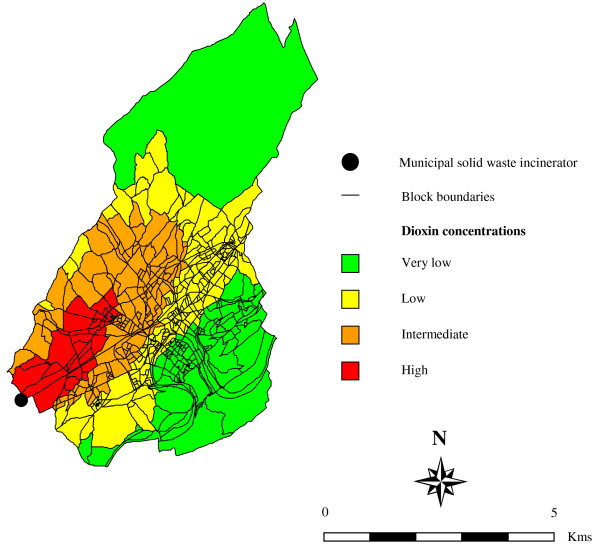
Modeled dioxin concentrations at the block level, on the North-East side of the municipal solid waste incinerator of Besançon, France.

The respective contours of these modeled ground-level air concentrations (classified as very low, low, intermediate, and high) were digitized and contoured onto the surface of the map with geographic information system (GIS) tools. We then overlaid a map of blocks onto the digital dioxin concentration map to attribute a dioxin concentration category to each of the 590 northeast blocks (provided half or more of their area was within a given contour) (Figure 1). From their respective residence block, we then obtained a risk field classification for each cancer patient and control. Regarding socioeconomic characteristics defined at the block group level (education, occupational social class and household-based indicators), we have already shown that they did not vary with dioxin exposure category (Table 1) [17].

**Table 1:** Socioeconomic characteristics (defined at the block group level) of dioxin exposure zones (Kruskall – Wallis tests) [17]

	Very low	Low	Intermediate	High	*p*-value
Persons with a high school diploma (%)	33.9	30.2	26.6	28.4	0.21
Women in labor force (%)	46.8	49.3	47.6	54.8	0.23
Workers in labor force (%)	16.6	24.1	26.5	23.2	0.08
Unemployed in labor force (%)	12.8	14.8	15.1	12.8	0.84
Single woman as head of household (%)	6.8	7.7	10.6	9.3	0.15
Owner-occupied houses (%)	34.8	32.1	29.1	36.1	0.88
Number of persons per dwelling (mean)	2.25	1.94	2.21	2.25	0.34
Single-family houses (%)	35.1	13.8	12.1	30.4	0.17

### Statistical analysis

We used conditional logistic regressions to calculate odds ratios (ORs) and 95% CIs for each level of dioxin exposure estimated from the dispersion model. A set of dummy variables was generated for this categorical scale variable using the lowest category as the reference group. Models were run with LogXact software (CYTEL Software Corporation, Cambridge, MA, USA).

## Results

From 1996 to 2002, a total of 434 invasive breast carcinomas was diagnosed in the northeast side of the city of Besançon, and 2170 population controls were therefore randomly selected.

These cases corresponded to an age-standardized (world) incidence rate of 81.4 per 100,000, to be compared to the age-standardized (world) incidence rate of 90.4 per 100,000 for France, as estimated in 2000 (comparative morbidity figure [CMF] = 0.90, 95% CI, 0.81–1.00), and the age-standardized (world) incidence rate of 76.9 per 100,000 for the Doubs region that comprises the city of Besançon (CMF = 1.06, 95% CI, 0.95–1.18).

All but 12 cases were histologically confirmed (97.23%). Duct NOS and lobular carcinomas were the most common histologic subtypes accounting for 79.26% and 11.75%, respectively.

**Figure 2:**
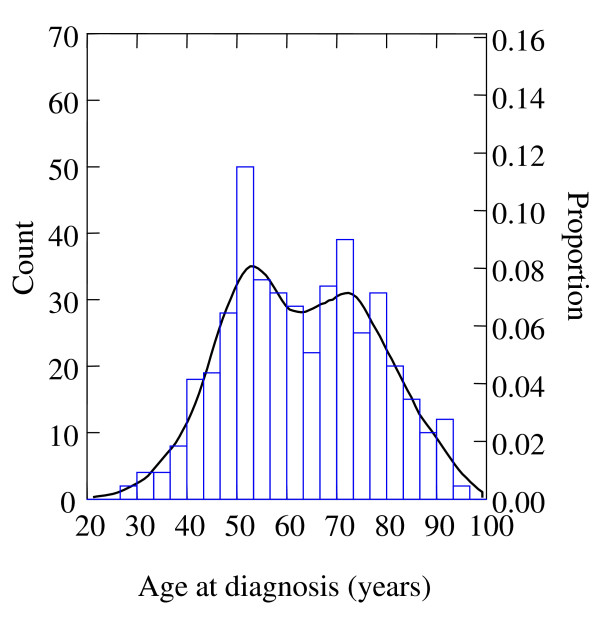
Age at diagnosis-histogram and density plot (or smoothed histogram) of invasive breast cancer (women residing on the North-East side of the municipal solid waste incinerator, Besançon, France, 1996–2002).

The age distribution at diagnosis for all breast cases combined showed a bimodal pattern with incidence peaks (or modes) near ages 50 and 70 years (Figure 2). This prompted us to run models separately for women aged 20–59 years, and women aged 60 years or older.

The distributions of these cancer patients by age bands and dioxin exposure categories are displayed in Table 2. Among women aged less than 60 years old, no increased or decreased risk was found for any dioxin exposure category. Conversely, for ages 60 years and over, women living in the highest exposed zone were 0.31 time less likely (95% CI, 0.08–0.89) to develop invasive breast carcinoma than women living in the very low emission area, with no relative risk estimate different from one for the other dioxin risk categories.

**Table 2:** Odds Ratio and 95% confidence interval of invasive breast cancer by age bands and dioxin exposure categories

Dioxin exposure	Cases	Controls	OR* (95% CI^†^)
*Women aged 20–59 years*			

Very low	41	225	1.0
Low	81	414	1.06 (0.72–1.56)
Intermediate	64	279	1.25 (0.82–1.89)
High	11	67	0.88 (0.43–1.79)

*Women aged 60 years and over*			

Very low	50	228	1.0
Low	111	558	0.90 (0.63–1.29)
Intermediate	72	339	0.96 (0.66–1.41)
High	4	60	0.31 (0.08–0.89)

* odds ratio

^† ^confidence interval

Excluding the 12 non-histologically confirmed cases did not alter the findings (results not shown).

## Discussion

Overall, our results do not provide evidence for an association between dioxins emitted from the MSWI and breast cancer risk among younger women, but show a decreased risk among older women residing in areas classified as highly exposed to dioxins.

Our study has a number of strengths. It is population-based and involves cases actively identified through multiple sources within a defined geographic area. Controls were randomly selected from a complete directory of people residing in the same area with a modest but relevant and reliable list of characteristics available at low cost (census data). The 5-to-1 matching procedure (usually considered to be a good trade-off regarding statistical power), kept constant across strata, produced fairly precise relative risk estimates, as reflected by the narrowness of the corresponding confidence intervals (Table 1).

The modeled ground-level concentrations represented the best available surrogates for past dioxin exposure measurements from the same source, given that no earlier measurements had been taken. By restricting our survey to the North East side of the MSWI, we were able to use dioxin exposure data based on sophisticated methods for modeling of emissions. GIS tools, in combination with dioxin soil measurements to validate the generated exposure metrics, allowed the classification of exposure across the study population, while avoiding the biases and limitations of self-reported exposures.

Regarding other occupational or environmental sources of exposure to dioxins, there are no adjacent industrial sources of combustion-effluents; highly polluting industries were replaced 2 decades ago by small-scale advanced technologies. No cement kilns, iron or steel works or foundries are located in this area. Other potential thermal and combustion sources, such as automobile exhausts and home heating, result in diffuse emissions. We have recently confirmed that the MSWI was indeed the single dominant point source of PCDD/Fs in this area [20].

Despite these strengths, our study has some limitations worth noting. Controls were residents in 1999, whereas cases were diagnosed between 1996 and 2002, introducing a time lag in the sampling for some matched sets. However, the population of Besançon appears to be stable over time for the age groups considered; 86% of the people over 40 years of age who lived in Besançon in 1999 were already residing in the city in 1990. Hence, the shortness of this time lag should not have affected the coverage of the target population, and if so, would bias the odds ratios towards 1.0.

This study is of mixed individual/ecological design with case and control residences linked to the dispersion map at the block level. Thus, although census blocks have a limited area (decreasing the distance between actual and surrogate locations) and were assigned one of four exposure levels prior to control sampling, the possibility of some non differential exposure misclassification cannot be ruled out.

### Confounding

Neither interviews nor medical data collection were done for the 2170 population controls. We only relied on census-based data, anonymous and parsimonious to protect privacy. We could not therefore verify that controls were "breast cancer free" (but such a misclassification would bias the odds ratio towards the null). Adjustment for age was imprecise, due to the width (20 years) of the age categories used for the control sampling. The lack of information pertaining to residence history and time-activity patterns limited our ability to ascertain the duration of exposure. Considering the long exposure-to-effect interval, in- and out-migrations may have occurred, inducing a potential misclassification. However, as migration is likely to be random with respect to disease status, it would result in a bias of risk estimates towards the null.

There are many known risk factors for breast cancer that we were unable to include in our analyses. In particular, women aged 60 years and above exposed to risk factors specific to postmenopausal women (body mass index, natural menopause, hormone therapy, educational level [2]) could be proportionally less abundant in the highest exposed zone. Unfortunately, we did not know the local distribution of these risk factors, and hence, whether they could explain some of the risk reduction observed in women aged 60 years and over and residing in the most exposed zone. Regarding socioeconomic level, a higher risk of breast cancer has been consistently reported for women having higher socioeconomic status or living in affluent communities. But, to confound our results, women aged 60 years and over living in the high exposure zone should be less well-off. In this respect, the similarities across bloc groups characterized by differing exposure levels are reassuring. However, for all the above-mentioned reasons, we cannot firmly exclude the possibility that residual confounding affected the reported odds ratios.

### Age distribution patterns

Our descriptive analysis of this population based data provides evidence for two main breast cancer populations, according to age at onset, mixed within breast cancer incidence overall. A shift to earlier age at diagnosis resulting from an increased compliance with screening mammography recommendations is unlikely, since the first breast cancer screening campaign in this area started late 2003.

One breast cancer type was mostly early-onset with peak incidence near age 50 years. The second breast cancer type was largely late-onset with its mode occurring at age about 70 years. This bimodal age incidence pattern has been observed worldwide, suggesting etiologic heterogeneity due to at least two biological subtypes or causal pathways [21]. In general, high-risk tumors are surrogates for bimodal breast cancer populations, shifted towards the early-onset mode near 50 years of age. Low-risk tumors are surrogates for bimodal breast cancer, weighted towards the late-onset mode at age 70 years. Moreover, bimodality in the general breast cancer population might be a reflection of early-onset of hereditary (or familial) versus late-onset non-hereditary (acquired or sporadic, and then more likely to be associated with environmental risk factors) breast cancer types. Additional etiologic clues could be provided by our contrasted results among women aged 60 years and over, the decreased risk in the most exposed zone being compatible with dioxin anti-estrogenic activity.

### Dose-response relationship

One striking feature lies in the absence of dose-response relationship but the presence of a possible threshold effect at the highest dioxin concentration category (associated with a decreased breast cancer risk). This threshold effect was also noticeable for NHL, but with an increased risk [17]. Unfortunately, as we used a ranking system rather than quantitative measurements to classify exposure levels we cannot be more precise about this threshold level.

### Comparison with other environmental studies

Our results stand in contrast to the findings from two previous studies. A mortality study conducted in Russia reported a higher overall risk of breast cancer (SMR = 2.1, 95% CI 1.6–2.7) among women living in Chapaevsk [22]. From 1967 to 1987, a chemical plant from this area produced hexachlorocyclohexane, resulting in intense dioxin contamination in the environment (air, soil, and drinking water). High concentrations of dioxins were detected in human milk and female workers' blood. However, no details are given on the quality, completeness, and disease classification of the mortality registration system, hampering the assessment of the strengths, weaknesses, and generalisability of this study.

Serum analysis of a subgroup of 981 women living in the high exposure zones around Seveso is also suggestive of an association between TCDD exposure and breast cancer risk [15]. Serum samples were collected within five years of the accident (1976) and analyzed for TCDD in 1996–98. Fifteen women reported having been diagnosed with breast cancer, and the diagnosis was confirmed by pathology in 13 cases. However, this study suffered some limitations, among which were the lack of increased incidence in the whole cohort, the improper follow-up of the sub-cohort and a RR that was no longer statistically significant after exclusion of the two non-confirmed cases.

Our study, on the other hand, is consistent with two different hospital-based case-control studies, that found no association between adipose levels of PCDD/Fs and breast cancer risk [14,23]. They were however limited by their small number of breast cancer cases (79 cases and 52 controls, 22 cases and 19 controls, respectively), the measurement of dioxin adipose concentrations at (or near) the time of diagnosis, their hospital-based (and not population-based) design, and the use of women undergoing surgery for benign breast conditions as the control group (which may result in over-matching).

## Conclusion

Before speculating that dioxin anti-estrogenic activity has greater effect on late-onset acquired breast cancer, we must envisage some residual confounding. Considering the inconclusive evidence from studies undertaken so far, future large-scale population based studies that include assessment of family history, breast cancer risk factors, environmental exposures, standardized histopathology reviews and molecular characterization are needed to lead to new insights into the association between environmental dioxin exposure and breast cancer risk.

## Abbreviations

Duct NOS: infiltrating duct carcinoma of no special type; GIS: geographic information system; ICD-O: International Classification of Diseases for Oncology; I-TEQ: international toxic equivalency factor; MSWI: municipal solid waste incinerator; PCDD: polychlorinated dibenzo-*p*-dioxins; PCDF: polychlorinated dibenzofurans; TCDD: 2,3,7,8-tetrachlorodibenzo-*p*-dioxin.

## Competing interests

The author(s) declare that they have no competing interests.

## Authors' contributions

JFV conceived and designed the study, and drafted the manuscript. MCC participated in the study design of the study, and carried out the statistical analysis. MH performed the geocoding, and participated in the geographical analysis. SG participated in the geographical analysis. BC carried out the control sampling, and helped interpret the findings. AD participated in the study design, and helped interpret the findings.
